# Ultrasonography-Guided Pigtail Catheter Drainage Versus Needle Aspiration for the Treatment of Liver Abscesses: A Comparative Study of Outcomes

**DOI:** 10.7759/cureus.85407

**Published:** 2025-06-05

**Authors:** Harsha S, Bhaskar Mallaiah, Sreenidhi G M

**Affiliations:** 1 General Surgery, Siddaganga Medical College and Research Institute, Tumkur, IND; 2 General Surgery, Kempegowda Institute of Medical Sciences, Bengaluru, IND

**Keywords:** abscess resolution, comparative study, liver abscess, minimally invasive intervention, percutaneous needle aspiration, pigtail catheter drainage, ultrasonography

## Abstract

Introduction

Liver abscesses remain a significant clinical challenge, particularly in tropical regions, despite advances in diagnostic and therapeutic approaches. This study aimed to compare the clinical outcomes of ultrasonography-guided pigtail catheter drainage (PCD) and percutaneous needle aspiration (PNA) in the management of liver abscesses.

Methods

A prospective observational study was conducted at a tertiary care center from October 2021 to March 2023. A total of 94 patients diagnosed with liver abscess were randomly assigned to two groups, with 47 patients each undergoing PCD and PNA. Baseline characteristics, clinical features, laboratory parameters, imaging findings, and treatment outcomes were recorded and analyzed using SPSS version 26 (IBM Inc., Armonk, NY).

Results

Right upper quadrant pain was the most frequent symptom, with a significantly higher occurrence in the PNA group (p = 0.041). The overall success rate was significantly higher in the PCD group (87.2%) compared to the PNA group (63.8%, p = 0.008). In the PNA group, single aspiration was sufficient in 18 patients (38.3%), whereas 29 patients (61.7%) required two or more aspirations. Time to achieve a 50% reduction in abscess cavity size was significantly shorter in the PCD group (p < 0.001). No significant differences were observed between the groups in hospital stay, time to clinical improvement, or adverse events. *Escherichia coli* was the most commonly isolated organism.

Conclusion

Ultrasonography-guided PCD is more effective than needle aspiration in managing liver abscesses, particularly for larger cavities. PCD enables continuous drainage, faster cavity resolution, and fewer repeat interventions, although PNA remains a simpler and cost-effective alternative in select cases.

## Introduction

A liver abscess is a localized collection of pus within the hepatic parenchyma, resulting from tissue necrosis, and typically containing leukocytes, necrotic debris, and microbial pathogens [1,2]. Liver abscesses account for approximately 15-20% of all intra-abdominal abscesses, representing a significant clinical entity, particularly in developing countries [1,3,4].

Despite their recognition dating back to Hippocratic times, liver abscesses can still present diagnostic and therapeutic challenges in low-resource settings, primarily due to limited access to imaging modalities. In regions where ultrasound and cross-sectional imaging are readily available, diagnosis, particularly of pyogenic liver abscesses, is generally straightforward and often guided by clinical acumen and a high index of suspicion, especially in endemic areas. Two major etiological types are identified: amoebic liver abscesses, predominantly caused by *Entamoeba histolytica*, and pyogenic liver abscesses, which are typically bacterial in origin. Globally, an estimated 40-50 million people are affected by amoebic liver abscesses, primarily in developing nations [4].

Under normal circumstances, the liver’s reticuloendothelial system effectively filters bacteria. However, abscesses can develop when this defense is overwhelmed - via the biliary tract, portal circulation, hepatic artery, or by contiguous spread from intra-abdominal infections such as appendicitis. In the Indian setting, biliary tract disease has emerged as a leading predisposing factor for pyogenic liver abscesses, which carry a high mortality rate of 80-100% if left untreated [5,6].

Over the past few decades, the management of liver abscesses has evolved from conventional surgical drainage to less invasive, image-guided percutaneous techniques. Ultrasonography (USG), computed tomography (CT), and fluoroscopy now aid in both diagnosis and therapeutic intervention. Among these, percutaneous drainage techniques, either via needle aspiration or catheter drainage, have demonstrated higher success rates and reduced morbidity compared to conservative medical management. While surgical drainage remains an effective option, especially in complex or refractory cases, image-guided percutaneous methods are now preferred as the first-line intervention in most clinical settings.

This study aims to compare the outcomes of USG-guided pigtail catheter drainage (PCD) and percutaneous needle aspiration (PNA) in patients with liver abscesses. Key outcome measures include duration of hospital stay, time to clinical improvement, reduction in abscess cavity size, and overall treatment success.

## Materials and methods

This prospective, observational, hospital-based study was conducted at KIMS Hospital, Bengaluru, over a period of 18 months from October 2021 to March 2023, after obtaining ethical clearance from the Institutional Ethics Committee (Ref: KIMS/IEC/D078/M/2021). The study aimed to compare clinical outcomes between USG-guided PCD and PNA in the management of liver abscesses.

The sample size was estimated based on a previous study by Bansal A et al. [7], which reported the mean number of days to clinical improvement as 5.5 ± 2.2 days in the PNA group and 4.2 ± 1.7 days in the PCD group. Considering these values, and assuming a two-sided significance level of 5% and a power of 80%, the required sample size was calculated to be 32 patients in each group using the mean comparison formula and MedCalc statistical software (MedCalc Software Ltd., Ostend, Belgium). Accounting for a 10% non-response or dropout rate, the sample size was increased to 36 per group, with a rounded total of 40 patients in each group. However, the final study included 47 participants per group, ensuring adequate statistical power for comparative analysis.

Inclusion criteria consisted of patients with clinically and radiologically confirmed liver abscess who provided informed consent. Patients were excluded if they had abscesses smaller than 5 cm in diameter, abscess volumes greater than 500 cc, prior intervention for liver abscess, ruptured abscesses, concomitant biliary tract malignancy, uncorrectable coagulopathy, or non-aspirable abscesses.

Simple randomization was performed using a computer-generated random number table to assign patients equally to the two groups: Group A underwent PNA, and Group B underwent PCD. Allocation was done in sealed opaque envelopes to ensure allocation concealment. Due to the nature of the interventions, blinding of the patient and proceduralist was not feasible; however, outcome assessment, data entry, and statistical analysis were performed by a blinded investigator to reduce potential bias.

All patients received initial empirical intravenous antibiotics comprising amoxicillin-clavulanate 1.2 g every 8 hours, metronidazole 500 mg every 8 hours, and gentamicin 5 mg/kg once daily, adjusted based on renal function and continued until culture sensitivity reports were available or clinical improvement was observed. Routine investigations, including liver function tests and coagulation profiles, were performed. Anemia was corrected with transfusions where necessary. All patients initially underwent USG for diagnosis and guidance of intervention. CT abdomen was selectively performed in cases where USG findings were inconclusive, the lesion was inaccessible or atypical, multiple abscesses were suspected, or in non-responders, to differentiate liver abscesses from other hepatic lesions such as hydatid cysts, simple cysts, or hemangiomas.

Liver abscesses were initially diagnosed using USG, which provided information on the size, location, and internal characteristics of the lesions. Pyogenic liver abscess was suspected based on clinical features such as fever, chills, and right hypochondrial tenderness, and was confirmed by positive bacterial growth in the aspirated pus. USG also served as the guiding modality for both procedures. All patients with clinical suspicion of liver abscess underwent USG within 6 hours of admission, regardless of the time of presentation. Emergency USG services were available 24/7, including night shifts, allowing for early diagnosis and planning of appropriate intervention. In our study, repeat aspirations were permitted in the PNA group based on clinical and imaging findings. Single aspiration was sufficient in 18 patients (38.3%), while 29 patients (61.7%) required two or more aspirations.

PCD was performed under ultrasound guidance using either the Seldinger technique, starting with an 18-gauge needle followed by guidewire and catheter insertion, or by directly inserting a 12 French trocar catheter. No major procedure-related complications were observed in either group during the study period. In the PCD group, catheter placement was guided by ultrasound, and patients were managed according to a standardized protocol. Daily monitoring of clinical status and drain output was performed. Repeat USG was conducted every 48-72 hours to assess cavity resolution and catheter position. The catheter was removed once the output decreased to less than 10 mL/day for two consecutive days, and ultrasound confirmed at least an 80% reduction in cavity size. Flushing or re-aspiration through the catheter was not routinely performed.

The outcome variables included the duration of hospital stay, time to clinical improvement (defined as the resolution of fever and right upper quadrant pain along with a reduction in total leukocyte count [TLC]), time taken for 50% reduction in abscess cavity size as assessed by USG, time to total or near-total resolution of the abscess cavity, and the overall treatment success rate. Treatment success was defined as either a reduction of ≥50% in cavity size or near-complete resolution on follow-up imaging, without the need for surgical intervention.

All data were compiled in Microsoft Excel (Microsoft Corp., Redmond, WA) and analyzed using SPSS version 26 (IBM Inc., Armonk, NY). Continuous variables were expressed as mean and standard deviation and compared using the independent-samples t-test. Categorical variables were compared using the chi-square test. A p-value of <0.05 was considered statistically significant.

## Results

The study included 94 subjects, with equal distribution in the PNA (n = 47) and PCD (n = 47) groups. The majority of patients [13 (27.7% each)] in both groups were aged between 41 and 50 years. The gender distribution was identical, with 36 (76.6%) male patients and 11 (23.4%) female patients in both groups (Table 1).

**Table 1:** Comparison of characteristics between the groups Data are expressed as mean ± SD for continuous variables and N (%) for categorical variables. Statistical tests used: Independent-samples t-test for continuous variables, chi-square test for categorical variables. Statistical significance is considered at p <0.05.

Variable	Group PNA (N, %)	Group PCD (N, %)	Test Statistic (χ²)	p-Value
<30 years	7 (14.8%)	3 (6.4%)	3.79	0.641
31-40 years	10 (21.3%)	12 (25.5%)		
41-50 years	13 (27.7%)	13 (27.7%)		
51-60 years	12 (25.6%)	11 (23.4%)		
>60 years	5 (10.6%)	8 (17.0%)		
Male	36 (76.6%)	36 (76.6%)	0.00	1.000
Female	11 (23.4%)	11 (23.4%)	0.00	1.000

In both groups, common risk factors identified included diabetes mellitus, biliary tract disease, and alcohol consumption. Diabetes was present in 21 patients (44.7%) in the PNA group and 19 patients (40.4%) in the PCD group. Biliary tract disease was noted in 11 (23.4%) and 14 (29.8%) patients in the PNA and PCD groups, respectively. A history of chronic alcohol use was reported in 18 patients (38.3%) in the PNA group and 20 (42.6%) in the PCD group. There were no statistically significant differences between the groups regarding these comorbidities (p > 0.05). Occupational data were collected but showed no consistent association in our population (Table 2).

**Table 2:** Risk factors and comorbidities Chi-square test (χ²) was used to compare categorical variables (presence or absence of risk factors such as diabetes mellitus, biliary tract disease, alcohol consumption, and hepatic disorders) between the two independent groups, PNA and PCD. A p-value greater than 0.05 indicates that there is no statistically significant difference between the groups for the given variable. PNA, percutaneous needle aspiration; PCD, pigtail catheter drainage.

Comorbidity/Risk Factor	Group PNA (n = 47)	Group PCD (n = 47)	χ²	p-Value
Diabetes mellitus	21 (44.7%)	19 (40.4%)	0.17	0.677
Biliary tract disease	11 (23.4%)	14 (29.8%)	0.54	0.462
Alcohol consumption	18 (38.3%)	20 (42.6%)	0.18	0.669
Known hepatic disorder	2 (4.3%)	3 (6.4%)	0.21	0.648

**Figure 1:**
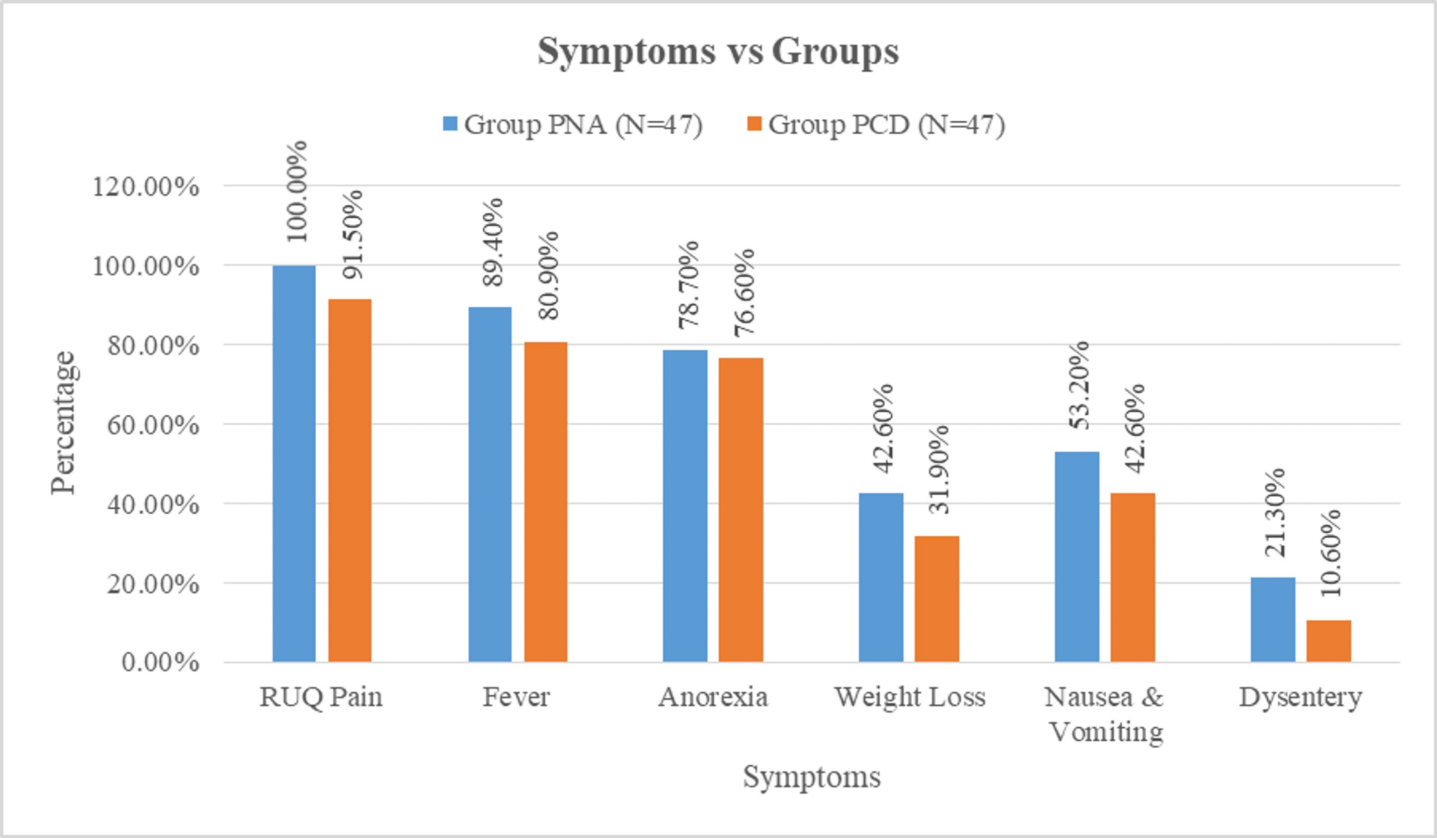
Comparison of symptoms between the groups PNA, percutaneous needle aspiration; PCD, pigtail catheter drainage; RUQ, right upper quadrant.

As illustrated in Figure 1, the most common presenting symptoms in both groups included right upper quadrant pain, fever, anorexia, and gastrointestinal disturbances. Right upper quadrant pain was reported by all patients in the PNA group and 91.5% of the PCD group, which was the only symptom showing a statistically significant difference between the groups (p = 0.041). Other symptoms, such as fever, nausea, vomiting, and weight loss, were comparable between the groups (Figure 1).

As summarized in Table 3, there were no statistically significant differences between the PNA and PCD groups with respect to key laboratory parameters, including TLC, serum bilirubin, and alkaline phosphatase (ALP). Other parameters, such as renal function tests, were within normal limits in most patients and did not differ significantly between groups. Inflammatory markers like CRP and procalcitonin, and nutritional markers like serum albumin were not consistently available for all patients and were therefore not included in the comparative analysis.

**Table 3:** Comparison of laboratory parameters between the groups Data are expressed as mean ± SD for continuous variables and N (%) for categorical variables. Statistical tests used: Independent-samples t-test for continuous variables, chi-square test for categorical variables. Statistical significance is considered at p <0.05. Organism names are italicized as per microbiological conventions. TLC, total leukocyte count; ALP, alkaline phosphatase; SD, standard deviation; mg/dL, milligrams per deciliter; IU/L, international units per liter; PNA, percutaneous needle aspiration; PCD, pigtail catheter drainage.

Parameter	Group PNA (Mean ± SD)	Group PCD (Mean ± SD)	Reference Range	Test Statistic (t)	p-Value
Total leukocyte count (×10³/mm³)	13.05 ± 5.57	13.46 ± 4.85	4-11	0.38	0.704
Serum bilirubin (mg/dL)	1.46 ± 1.97	1.88 ± 0.38	0.3-1.2	1.44	0.154
ALP (IU/L)	185.72 ± 156.96	176.73 ± 89.02	44-147	0.34	0.733
Culture positivity, n (%)	21 (44.7%)	23 (48.9%)	–	χ² = 0.13	0.714
Most common organism	Escherichia coli	Escherichia coli	–	–	–
Other organisms isolated	*Klebsiella* spp.	Staphylococcus aureus	–	–	–

Further investigations, including amoebic serology, the presence of anchovy sauce-like pus, and culture of aspirated material, showed no significant differences between the groups. Culture was positive in 21 patients (44.7%) in the PNA group and 23 patients (48.9%) in the PCD group, with the majority of specimens showing no bacterial growth. *Escherichia coli* was the most commonly isolated organism in both groups, followed by *Klebsiella* in the PNA group and *Staphylococcus aureus* in the PCD group. These differences were not statistically significant.

Microbiological culture of aspirated pus was positive in 21 patients (44.7%) in the PNA group and 23 patients (48.9%) in the PCD group. The most commonly isolated organism in both groups was *Escherichia coli*, followed by *Klebsiella* species in the PNA group and *Staphylococcus aureus* in the PCD group. A detailed distribution of organisms is presented in Table 4.

**Table 4:** Organism breakdown Values are presented as number of patients (%); culture-negative samples are included for completeness.

Organism Isolated	PNA Group (n = 47)	PCD Group (n = 47)	Total (N = 94)
Escherichia coli	9 (19.1%)	11 (23.4%)	20 (21.3%)
*Klebsiella* spp.	6 (12.8%)	4 (8.5%)	10 (10.6%)
Staphylococcus aureus	2 (4.3%)	5 (10.6%)	7 (7.4%)
Pseudomonas aeruginosa	2 (4.3%)	1 (2.1%)	3 (3.2%)
Polymicrobial	1 (2.1%)	2 (4.3%)	3 (3.2%)
No growth	27 (57.4%)	24 (51.1%)	51 (54.3%)

With regard to abscess characteristics, 31 patients (65.9%) in the PNA group and 30 (63.8%) in the PCD group had pyogenic abscesses, while amoebic abscesses were found in 16 (34.1%) and 17 (36.2%) patients, respectively. On USG, the right lobe was the most common location (80.9% in PNA vs. 70.2% in PCD), followed by the left lobe (19.1% in both groups), while five patients (10.6%) in the PCD group had bilobar involvement. Multiple abscesses were observed in 11 patients (23.4%) in the PNA group and 13 patients (27.7%) in the PCD group, with no significant difference between groups (Table 5). 

**Table 5:** Comparison of details of abscess between the groups Data are expressed as N (%). Statistical tests used: Chi-square test. Significance threshold is set at p <0.05.

Variable	Group PNA (N, %)	Group PCD (N, %)	Test Statistic (χ²)	p-Value
Pyogenic	31 (65.9%)	30 (63.8%)	0.01	0.928
Amoebic	16 (34.1%)	17 (36.2%)		
Right lobe	38 (80.9%)	33 (70.2%)		
Left lobe	9 (19.1%)	9 (19.1%)		
Both lobes	0 (0.0%)	5 (10.6%)	5.35	0.069

Clinical variables were compared between the groups. The mean abscess volume was similar (425.67 ± 253.67 mL in PNA vs. 429.21 ± 225.48 mL in PCD). The mean hospital stay was 9.90 ± 7.43 days for PNA and 11.33 ± 7.61 days for PCD. Time to clinical improvement was 4.13 ± 2.16 days in the PNA group and 3.44 ± 2.11 days in the PCD group. The mean time to a 50% reduction in the abscess cavity size was significantly shorter in the PCD group (3.63 ± 1.54 days) compared to the PNA group (5.55 ± 1.22 days, p < 0.001). No significant differences were found in the time to near-total resolution (11.01 ± 3.41 weeks in PNA vs. 11.23 ± 2.62 weeks in PCD) or in the duration of intravenous antibiotic therapy (8.82 ± 2.42 days in PNA vs. 9.57 ± 2.98 days in PCD) (Table 6).

As shown in Table 6, most clinical outcome parameters, such as abscess volume, hospital stay, time to clinical improvement, and duration of intravenous antibiotic therapy, were comparable between the two groups. However, the time to achieve a 50% reduction in the abscess cavity size was significantly shorter in the PCD group compared to the PNA group (p < 0.001), suggesting faster radiological response with catheter drainage.

**Table 6:** Comparison of clinical variables between the groups Continuous variables were presented as mean ± standard deviation and analyzed using the independent-samples t-test. A p-value <0.05 was considered statistically significant. Statistically significant values are marked with an asterisk (*).

Subjects (N = 94)	Group PNA (N = 47)		Group PCD (N = 47)		p-Value
	Mean	SD	Mean	SD	
Volume of abscess cavity	425.67	253.67	429.21	225.48	0.943
Duration of hospital stay	9.90	7.43	11.33	7.61	0.359
Duration of clinical improvement	4.13	2.16	3.44	2.11	0.120
Duration till 50.0% decrease in size of cavity	5.55	1.22	3.63	1.54	<0.001*
Duration till total/near-total resolution of cavity	11.01	3.41	11.23	2.62	0.726
Duration of intravenous antibiotics	8.82	2.42	9.57	2.98	0.183

Regarding complications, three patients (6.4%) in the PCD group experienced adverse events, including recurrence and moderate to severe pain that required additional intravenous analgesia. No procedural complications or interventions such as repositioning or surgical management were needed. No complications were observed in the PNA group. This difference, however, was not statistically significant. The overall success rate was significantly higher in the PCD group (41 patients, 87.2%) compared to the PNA group (30 patients, 63.8%) (p = 0.016), indicating greater clinical effectiveness of catheter drainage (Table 7).

**Table 7:** Comparison of final outcome between the groups Data are expressed as N (%). Statistical tests used: chi-square test. Significance threshold is set at p <0.05.

Outcome	Group PNA (N, %)	Group PCD (N, %)	Test Statistic (χ²)	p-Value
Success	30 (63.8%)	41 (87.2%)	5.76	0.016
Failure	17 (36.2%)	6 (12.8%)		

## Discussion

In the present study, the majority of patients, 13 (27.7%), belonged to the 41-50 years age group, with ages ranging from 16 to 72 years and a mean age of 47.1 years. This age distribution is consistent with the findings from studies conducted in Bihar and Navi Mumbai, where liver abscesses were more common in the third and fourth decades of life [8,9].

A significant male predominance was observed, with 36 patients (76.6% of cases) being male patients. This aligns with the findings from other Indian studies, including one from Bihar (80%) and another from Navi Mumbai (92%) [8,9]. The higher incidence in male patients may be attributed to lifestyle factors such as alcohol consumption, occupational exposure, hormonal influences, and delayed health-seeking behavior, which increase the susceptibility to liver abscess.

Right upper quadrant pain was universally present in patients treated with PNA and in 43 patients (91.5%) of those treated with PCD. Fever was more frequently observed in the PNA group. These findings are consistent with the clinical profile reported in a study from Bihar, where fever (88%), right upper quadrant pain and tenderness (93%), and hepatomegaly (80%) were predominant clinical features [8].

In our study, positive amoebic serology was noted in 16 patients (34.0%) who underwent PNA and 14 patients (29.8%) who underwent PCD. Positive pus culture was reported in 21 patients (44.7%) who underwent PNA and 19 patients (40.9%) who underwent PCD. These findings are notably higher than those from a study conducted in Madhya Pradesh, which reported a culture positivity rate of only seven patients(14.7%) [10].

The most commonly isolated pathogen was *Escherichia coli* (18.0%), followed by *Klebsiella* (12.7%), with nearly half the cases showing no bacterial growth. These results align with the data from Bihar and Madhya Pradesh [8,10]. However, a study from Navi Mumbai reported *Klebsiella* as the most frequent isolate [9]. These differences likely reflect regional microbiological variations.

Our findings revealed a higher overall success rate with PCD in 41 patients (87.2%), compared to 30 patients (63.8%) with PNA, indicating the superior efficacy of PCD. These results are consistent with those reported in the studies from Navi Mumbai and Madhya Pradesh [9,10]. A randomized controlled trial by Rajak CL et al. also demonstrated better outcomes with PCD [11]. In contrast, a study by Yu SC et al. in 2004 found no significant difference in the success rates between the two techniques [12].

In our study, repeat aspirations were performed in the PNA group based on clinical response and follow-up USG findings. Only 38.3% of patients improved with a single aspiration, while 44.7% required two aspirations and 17.0% needed three or more attempts. This pattern closely mirrors the findings by Yu SC et al., who reported that 41% of patients required one aspiration, 41% required two, and 18% needed three or more. These data reinforce the point that PNA often necessitates multiple sessions, which may contribute to its relatively lower success rate and favor the use of PCD for more definitive and continuous drainage.

No statistically significant difference was found in time to clinical improvement between groups in our study, echoing the findings of Singh S et al. [13]. However, a study from Madhya Pradesh reported a significant difference favoring faster recovery with PCD [10]. Such variations may be attributed to differences in sample size, abscess characteristics, comorbidities, severity of infection, procedural skill, timing of intervention, and clinical improvement criteria.

As expected, our study found that the time to 50% reduction in abscess size was significantly shorter in the PCD group, consistent with the findings by Kumar RV et al. and Dubhashi SP et al., who also reported faster cavity resolution with catheter drainage [9,10].

The mean duration of hospital stay was slightly longer in the PCD group (11.33 days) than in the PNA group (9.90 days), though the difference was not statistically significant. This is in agreement with reports from Dubhashi SP et al. and Singh S et al., whereas Kumar RV et al. found shorter hospital stays with PNA [9,10,13].

The mean duration of intravenous antibiotic therapy was 9.57 days in the PCD group and 8.82 days in the PNA group, with no significant difference noted. This is consistent with the findings from Singh S et al. [13]. Similarly, the time to complete or near-complete resolution of the abscess cavity was comparable between the two groups (11.01 weeks in PNA vs. 11.33 weeks in PCD).

In terms of complications, no adverse events were observed in the PNA group. In the PCD group, three patients (6.4%) experienced complications, including moderate to severe pain and radiological recurrence of the abscess cavity. The pain was more than expected post-procedural discomfort and required stepwise escalation of analgesia, including intravenous medications. The recurrence involved reappearance of abscess on follow-up ultrasound after initial clinical improvement and was managed with antibiotics, repeat imaging, and, in one patient, repeat PCD. Although these events were clinically manageable and did not result in prolonged hospitalization or mortality, they highlight the need for careful follow-up after catheter-based interventions. This complication rate was not statistically significant compared to the PNA group but is consistent with the previous reports by Baek SY et al. and Giorgio A et al., who also noted a lower incidence of complications with needle aspiration [14,15]. Importantly, no mortality was recorded in either group during the study period, underscoring the overall safety of both procedures.

This study has certain limitations that should be acknowledged. First, it was conducted at a single tertiary care center, which may limit the generalizability of the findings to other settings with different patient populations or healthcare infrastructures. Second, although patients were randomly allocated to the PNA and PCD groups, the study was not blinded, and proceduralists were aware of the intervention being performed, which could introduce performance or observer bias. Third, microbiological confirmation was limited, as nearly half of the pus culture samples showed no growth, potentially affecting the accuracy of organism-specific analysis. Additionally, follow-up was based primarily on ultrasonographic resolution and clinical observation without long-term assessment of recurrence or late complications. Finally, while the sample size was adequate for detecting differences in primary outcomes, it may not have been powered to detect differences in less frequent events such as complications or mortality.

## Conclusions

This study demonstrates that USG-guided PCD is a more effective modality than PNA in the management of liver abscesses. PCD was associated with a significantly higher overall success rate and a shorter time to 50% reduction in abscess cavity size. While PNA remains a simpler and cost-effective option, especially in select patients, PCD offers the advantage of continuous drainage and reduced need for repeat interventions. Further studies stratifying outcomes by abscess size and complexity would help guide individualized treatment decisions.
